# Supplementary material for: Content-rich biological network constructed by mining PubMed abstracts
Source: BMC Bioinformatics. 2004 Oct 8;5:147. doi: 10.1186/1471-2105-5-147 (PMC528731; doi:10.1186/1471-2105-5-147)
Supplement: Additional File 5 — The original Chilibot query results of the term "long-term potentiation (LTP)" and 22 other terms, limiting the latest references analyzed to the years 1990, 1995, 2000, and 2004. [file 1471-2105-5-147-S5.bz2 › chilibotAdditionalFile5/ltp2000/html/NMDA_TAU.html]

 


 **NMDA** and **TAU** 
  
Found 75 abstracts in PubMed,  **30 abstracts were retrieved and analyzed**.  


---

 Search Google  |
 PDF files only 
|  EDU domain only 

---

**Interactive relationship** (e.g. stimulation, inhibition, etc)

- Modifications of neuronal phosphorylated  **tau**  immunoreactivity induced by  **NMDA**  toxicity.  Ref: 9147412 Mol Chem Neuropathol, 1996
- Stimulation with 100 microM N methyl D aspartate  **NMDA**  increased extracellular  **Tau**  in control rats by 27 % and in TAA treated rats by as much as 250%.  Ref: 10213478 J Neurosci Res, 1999
- Cultured neurons expressing phosphorylated  **tau**  are more resistant to apoptosis induced by  **NMDA**  or serum deprivation.  Ref: 9105678 Brain Res Mol Brain Res, 1997
- A 15 min pulse with 100 microM  **NMDA**  induced an increase in the levels of  **tau**  proteins in NT2 N cells.  Ref: 9535117 Neurosci Lett, 1998
- The 10 90% rise times of the pure  **NMDA**  EPSCs were slower 16 vs 10 msec, and the decay  **tau**  values were faster  **tau**  4 vs 42 msec.  Ref: 9698343 J Neurosci, 1998
- Both  **NMDA**  and AMPA kainate receptors are involved in this  **tau**  gene overexpression.  Ref: 9260742 J Neurosci Res, 1997
- The percentages of apoptotic neurons expressing phosphorylated  **tau**  AT8 immunoreactivity are comparable in control and  **NMDA**  exposed cultures 7.5 1.9 and 6.9 1.9%, respectively.  Ref: 9105678 Brain Res Mol Brain Res, 1997
- In cell attached patches,  **NMDA**  evoked currents that had an apparent mean open time  **tau**  o of 3.26 ms.  Ref: 9105235 Anesthesiology, 1997
- The results showed that 1 extracellular Gln was low whereas Glu and  **Tau**  were high during infusion of  **NMDA**  0.5 1.0 mM or high K 80 mM in the hippocampus and ventrobasal thalamus, 2 hippocampal extracellular Glu, Gln and  **Tau**  were increased in response to the Glu uptake inhibitor, L trans pyrrolidine dicarboxilic acid tPDC, 0.5 3.0 mM, in a concentration dependent manner, 3 high K induced increase of extracellular Glu was partially blocked by the addition of 10 mM CoCl 2 with the high K dialysate in the hippocampus.  Ref: 10812202 Neurochem IntNeurochem Int, 1997
- Intrathecal  **NMDA**  faciliated the effects of BIC and STR on TEA score and spinal release of Glu and  **Tau** .  Ref: 8725598 Masui, 1996
- Perfusion with the  **NMDA**  receptor antagonist 3 R 2 carboxypiperazin 4 yl propyl 1 phosphonic acid 1 mM or the AMPA kainate receptor antagonist dinitroquinoxaline dione DNQX 1 mM attenuated the increases produced by PDC 4 mM on DA, GABA, and  **Tau** , and decreases in DOPAC and HVA.  Ref: 9326276 J Neurochem, 1997

**Parallel relationship** (e.g. studied together, co-existance, homology, etc.)

- An  **NMDA**  receptor antagonist, 2 amino 5 phosphonopentanoic acid 200 microM, blocked SRIF 100 nM evoked Asp, Glu,  **Tau** , and GABA release and reduced that of DA.  Ref: 9523593 J Neurochem, 1998
- In this study, using confocal laser microscopy and immunoblot analysis, we report that acute 500 mu M for 15 min or chronic 20 mu M for 16 h N methyl D aspartate  **NMDA**  neuronal toxicities modify the immunoreactivity of phosphorylated  **tau** .  Ref: 9147412 Mol Chem Neuropathol, 1996
- At the opposite, the percentage of apoptotic neurons expressing de phosphorylated  **tau**   **tau**  1 immunolabelings is dramatically increased in  **NMDA**  treated cultures X 2.3 of controls.  Ref: 9105678 Brain Res Mol Brain Res, 1997
- The increase of K or  **NMDA**  dependent  **Tau**  release may reflect improved cell volume regulation and neuroprotection and contribute to attenuation of neurologic symptoms in rats with liver failure.  Ref: 10213478 J Neurosci Res, 1999
- **tau**  67 vs 357 msec than  **NMDA**  EPSCs at mixed synapses.  Ref: 9698343 J Neurosci, 1998
